# Plague Epidemic in the Kingdom of Naples, 1656–1658

**DOI:** 10.3201/eid1801.110597

**Published:** 2012-01

**Authors:** Silvia Scasciamacchia, Luigina Serrecchia, Luigi Giangrossi, Giuliano Garofolo, Antonio Balestrucci, Gilberto Sammartino, Antonio Fasanella

**Affiliations:** Istituto Zooprofilattico Sperimentale of Puglia and Basilicata, Foggia, Italy (S. Scasciamacchia, L. Serrecchia, L. Giangrossi, G. Garofolo, A. Fasanella);; University Federico II, Naples, Italy (A. Balestrucci, G. Sammartino)

**Keywords:** Yersinia pestis, plague, epidemic, ancient, Kingdom of Naples, Italy, teeth, dental pulp, bacteria

**To the Editor**: In 1656, an epidemic of plague occurred in the Kingdom of Naples, Italy. Earlier the disease had spread from Algiers to Spain; in June 1647, it appeared in Valencia, and in the spring of 1648, it appeared in Aragon and several other Spanish areas of Valencia, Andalusia, and Catalonia. In 1652, plague had spread to Sardinia and then to the cities and territories of Naples, Rome, and Genoa. Within the Kingdom of Naples, plague first reached the town of Naples in the spring of 1656. Despite measures restricting population movement, by the summer of 1656, the disease had reached several provinces in southern Italy (*1**,**2*).

Historical records indicate that the epidemic in Barletta, in southern Italy, developed after the arrival of a ship from Naples. On May 26, 1656, the ship Sant’ Andrea arrived from Naples at the port of Barletta. However, after sanitary inspection, the ship was prevented from landing and obliged to depart, but this measure was not sufficient to prevent the disease from entering the port. The Barletta epidemic peaked in October, after which the number of cases diminished; and on June 22, 1657, Barletta was declared free of plague. Of this city’s original population of 20,000, the disease killed 7,000–12,000 persons. It is hypothesized that throughout the Kingdom, the plague killed ≈1,250,000 persons (*1**,**2*).

Since the 14th century, noble families of Barletta had been buried in tombs in underground tunnels of Sant’ Andrea church. During restoration of the church in 2009, more underground tunnels containing many skeletons were discovered. It has been hypothesized that the church had also been used as a cemetery during the plague epidemic. During an inspection of the skeletons, 5 skulls of young persons were identified and collected. For a negative control, the skull of a person buried in a tomb before the epidemic was also collected.

The skulls were radiographed to identify unerupted teeth (Figure), which were then aseptically extracted. After classification, each tooth was cut along a sagittal line to uncover the dental pulp, which was then hydrated in sterile phosphate-buffered saline (pH 7.2) for 48 h at 37°C. The DNA was extracted by using DNAeasy Blood and Tissue Kits (QIAGEN, Hilden, Germany) and by modifying the first step, which was conducted overnight at 56°C with 600 μL of ATL buffer (QIAGEN) and 50 μL of proteinase K. To verify the presence of inhibiting substance, the control DNA extracts were screened by using a PCR for human mitochondrial DNA (*3*).

**Figure:**
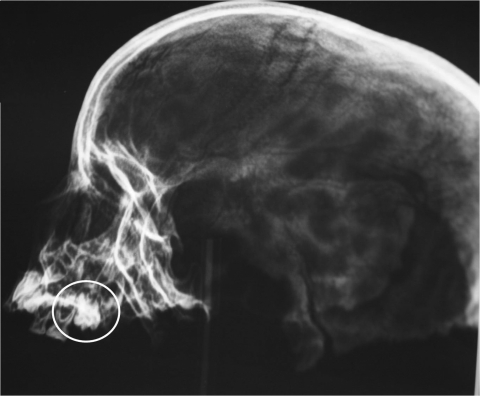
Radiograph of skull found under Sant’ Andrea church in Barletta, Italy, in 2009, showing unerupted teeth (circled) that were later extracted aseptically.

To investigate the cause of the deaths, we adopted a PCR suicide method and searched for *Yersinia pestis*. We amplified the *pla* gene for *Y. pestis* by using Sybr green PCR in real time with a modification of a previous protocol (*4*) coupled with conventional PCR according to Drancourt et al. (*5*). Conventional PCRs were adopted for *Bacillus anthracis* by targeting the *pag* and *capC* genes (*6*) and for *Salmonella enterica* serovar Typhi by targeting the *narG* gene (*7*). To prevent cross-contamination, we conducted all PCRs with a negative control and in the absence of positive controls. Melting curve analysis and agarose gel electrophoresis of PCR products indicated suspected positive samples. All amplicons relative to conventional and suicide PCR were submitted for sequencing analysis confirmation. The negative DNA was reanalyzed to confirm the results.

From the 26 dental pulp samples analyzed from the 5 skulls of young persons, 7 samples were positive for the *pla* gene of *Y. pestis* by the Sybr green real-time PCR, and 2 of these were positive for this gene by conventional PCR. All were negative for *B. anthracis* and *S. enterica* ser. Typhi. GenBank BLAST (www.ncbi.nlm.nih.gov/blast/Blast.cgi) results of the 2 sequenced amplicons found a 100% match with the reference sequences (GenBank accession no. AL109969.1); query coverage was 100%. The sequences obtained were deposited in the GenBank sequence database under accession nos. JN208020–1.

In conclusion, the confirmed finding of DNA of *Y. pestis* in 2 skeletons and suspected finding in the remaining 3 suggests that these persons died of plague during the 1656–1658 epidemic in southern Italy. Although it has not been universally agreed upon, several studies have confirmed that the agent of 16th to 18th century “plague” epidemics in Europe were caused by *Y. pestis*. Different methods have documented *Y. pestis* as the agent in 10 Black Death burial sites scattered over 5 countries (*8*). In northern Italy, the presence of *Y. pestis* has been confirmed in Venice (14th–17th centuries) (*8*), Genoa (Bastione dell’Acquasola) (14th century) (*9*), and Parma (16th–17th centuries) (*10*). This study confirms that the plague that infected the Kingdom of Naples, which spanned almost all of southern Italy, was also caused by *Y. pestis*.
